# Peptain-1 blocks ischemia/reperfusion-induced retinal capillary degeneration in mice

**DOI:** 10.3389/fncel.2024.1441924

**Published:** 2024-08-01

**Authors:** Mi-Hyun Nam, Armaan Dhillon, Rooban B. Nahomi, Noelle L. Carrillo, Clarinda S. Hougen, Ram H. Nagaraj

**Affiliations:** ^1^Department of Ophthalmology, UCHealth-Sue Anschutz-Rodgers Eye Centre, School of Medicine, University of Colorado, Aurora, CO, United States; ^2^Department of Radiology, UCHealth University of Colorado Hospital, Aurora, CO, United States; ^3^Department of Pharmaceutical Sciences, Skaggs School of Pharmacy and Pharmaceutical Sciences, University of Colorado, Aurora, CO, United States

**Keywords:** neurovascular degeneration, proinflammatory cytokines, peptain-1, human retinal endothelial cells, apoptosis, ischemia/reperfusion injury

## Abstract

**Introduction:**

Neurovascular degeneration results in vascular dysfunction, leakage, ischemia, and structural changes that can lead to significant visual impairment. We previously showed the protective effects of peptain-1, a 20 amino acid peptide derived from the αB-crystallin core domain, on retinal ganglion cells in two animal models of glaucoma. Here, we evaluated the ability of peptain-1 to block apoptosis of human retinal endothelial cells (HRECs) *in vitro* and retinal capillary degeneration in mice subjected to retinal ischemia/reperfusion (I/R) injury.

**Methods:**

HRECs were treated with either peptain-1 or scrambled peptides (200 μg/mL) for 3 h and a combination of proinflammatory cytokines (IFN-γ 20 ng/mL + TNF-α 20 ng/mL+ IL-1β 20 ng/mL) for additional 48 h. Apoptosis was measured with cleaved caspase-3 formation via western blot, and by TUNEL assay. C57BL/6J mice (12 weeks old) were subjected to I/R injury by elevating the intraocular pressure to 120 mmHg for 60 min, followed by reperfusion. Peptain-1 or scrambled peptide (0.5 μg) was intravitreally injected immediately after I/R injury and 7 days later. One microliter of PBS was injected as vehicle control, and animals were euthanized on day 14 post-I/R injury. Retinal capillary degeneration was assessed after enzyme digestion followed by periodic acid–Schiff staining.

**Results:**

Our data showed that peptain-1 entered HRECs and blocked proinflammatory cytokine-mediated apoptosis. Intravitreally administered peptain-1 was distributed throughout the retinal vessels after 4 h. I/R injury caused retinal capillary degeneration. Unlike scrambled peptide, peptain-1 protected capillaries against I/R injury. Additionally, peptain-1 inhibited microglial activation and reduced proinflammatory cytokine levels in the retina following I/R injury.

**Discussion:**

Our study suggests that peptain-1 could be used as a therapeutic agent to prevent capillary degeneration and neuroinflammation in retinal ischemia.

## Introduction

The neurovascular unit of the retina consists of neural cells, endothelial cells, pericytes, and glial cells such as microglia, Müller glia, and astrocytes (Oshitari, 2023). The delicate balance among these cells can be disrupted in diabetic conditions, which can have severe effects on vision (Brucklacher et al., 2008; Mohite et al., 2023; Oshitari, 2023). It is believed that these disruptions begin early in the diabetic retina, often before overt pathological changes become evident (Stitt et al., 2016). During this early phase, impaired retinal blood flow, capillary occlusion, and hypoxia contribute to the ischemic conditions that drive the development of diabetic retinopathy (DR) (Mohite et al., 2023). Consequently, protecting the neurovascular unit from these early ischemic insults may be crucial in preventing the progression of DR to more severe stages of vision loss.

Globally, an estimated 463 million adults had diabetes in 2019, with this number projected to increase to 700 million by 2045 (Lapertosa et al., 2019; Khan et al., 2020). According to the 2020 National Diabetes Statistics Report, the combined prevalence of type 1 and type 2 diabetic patients over the age of 18 in the US was 31 million as of 2018 which accounts for 13.0% of the adult population (Centers for Disease Control and Prevention, 2021). DR is one of many devastating sequelae of long-term hyperglycemia but is the leading cause of visual impairment in working-class adults in the US (Morello, 2007). It is thus of the utmost importance that steps are taken to address this quality-of-life-limiting complication (Xu et al., 2018).

Inflammation and oxidative stress are major factors in retinal capillary cell apoptosis in DR. It has previously been established that proinflammatory cytokines, including interferon-γ (IFN-γ), tumor necrosis factor-α (TNF-α), monocyte chemoattractant protein-1 (MCP-1), interleukin-1β (IL-1β), and interleukin-6 (IL-6), are upregulated in the diabetic retina as a consequence of chronic hyperglycemia (King, 2008; Zhou et al., 2012; Nahomi et al., 2014; Gui et al., 2020). The chronic inflammatory state of diabetes triggers both thickening of the capillary basement membrane and apoptosis of retinal neuronal cells, capillary pericytes, and endothelial cells (Shin et al., 2014; Mrugacz et al., 2021). Disruption of the regular capillary structure in turn leads to the formation of microaneurysms, capillary occlusion, and retinal ischemia (Frank, 1984; Shin et al., 2014; Fu et al., 2016; Lopez-Luppo et al., 2017). Additionally, retinal capillary endothelial cells are highly responsive to inflammatory cytokines such as IFN-γ, TNF-α, and IL-1β, which induce increased retinal capillary permeability and transcapillary migration of immune cells, further promoting the inflammatory cascade (Rübsam et al., 2018). Capillary damage and subsequent retinal ischemia lead to upregulation of vascular endothelial growth factor (VEGF), which is a target in many therapeutic interventions (Gupta et al., 2013). Further progression of DR, mediated by the upregulation of VEGF, will eventually lead to neovascularization, breakdown of the blood-retina barrier, macular edema, retinal detachment, and eventual vision loss (Eshaq et al., 2017; Homme et al., 2018). Unfortunately, 30% of patients with DR continue to progress despite anti-VEGF therapy, reinforcing the need for additional treatment options (Zhao and Singh, 2018).

At present, there are only limited and invasive treatments available for the advanced stages of DR and there is an urgent need to identify molecular targets in order to treat the earlier stages of the disease. Perhaps the most intriguing prospect is addressing retinal ischemia. It is well established in the literature that ischemic retinopathy occurs early on in diabetic retinopathy (Mohite et al., 2023) and that downstream sequelae of this ischemia, such as neovascularization, is central to the pathogenesis of DR (Hammes et al., 2002; Osborne et al., 2004; Noda et al., 2012). Thus, a minimally invasive therapeutic aimed at addressing these early ischemic changes could pay untold dividends for patients at risk of progressing to blindness from DR.

One such target that has shown some promise comes from the crystallin group of proteins. Crystallins are the major structural proteins in the lens and are subdivided into 3 families, namely, α-, β-, and γ-crystallins. The α-crystallins, αA- and α B-, are members of the small heat shock protein (sHSP) family, which are both ATP-independent molecular chaperones and antiapoptotic (Webster et al., 2019). They protect against stress-mediated apoptosis in several cell types, including the lens epithelium and retinal cells (Andley et al., 2000; Andley, 2007; Kannan et al., 2012). α-Crystallin has been shown to be highly expressed in retinal ganglion cells and Müller glial cells in patients with DR (Rübsam et al., 2018) suggesting a potential role in the cellular stress response. However, its functional activity in the diabetic retina remains uncertain due to posttranslational modifications occurring under hyperglycemic conditions (Losiewicz and Fort, 2011; Karumanchi et al., 2015). Thus, introducing a fully operational α-crystallin or mimicking the function of the protein could be a promising therapeutic strategy. Such an intervention could help protect retinal cells during the early stages of DR when ischemic changes are highly prevalent. Sharma and colleagues previously identified a 20 amino acid peptide from the core domain of αB-crystallin that has similar chaperone activity to its parent protein (Bhattacharyya et al., 2006). This peptide was dubbed “peptain-1”. We have previously shown that injections of peptain-1 can block apoptosis of lens epithelial cells, inhibit cataract formation, and protect retinal ganglion cells in animal models of glaucoma (Nahomi et al., 2013b; Stankowska et al., 2019; Nam et al., 2022). Here, we tested the effect of peptain-1 against proinflammatory cytokine-induced death of human retinal endothelial cells (HRECs) *in vitro* and ischemia/reperfusion (I/R) injury-mediated retinal capillary degeneration, aiming to demonstrate the efficacy of peptain-1 treatment under ischemic conditions such as those found in early DR (Osborne et al., 2004). Our results show that peptain-1 inhibits cytokine-mediated death of HRECs and retinal capillary degeneration caused by I/R stress in mice.

## Materials and methods

### Peptides

All peptides used in this study were obtained from Peptide 2.0 (Chantilly, VA) and were 95–99% pure. Their expected molecular weights were confirmed by liquid chromatography-mass spectrometry. The amino acid sequences of peptides used in this study are shown below:

Peptain-1: DRFSVNLDVKHFSPEELKVK

Peptain-1 with an additional cysteine residue (Peptain-1-Cy5): DRFSVNLDVKHFSPEELKVKVC

Scrambled peptide-1 (Scrb-1): SLKEKRNFDVSEVKHVL FVDP

Scrambled peptide-2 (Scrb-2): FEPSVRFSKVDHLVKEN DLVK

The scrambled peptides had 21 amino acids, unlike 20 in peptain-1, with an extra valine residue; this was based on the original report by Sharma’s group of the chaperone peptide with 21 amino acid residues (DRFSVNLDVKHFSPEELKVKV) (Bhattacharyya et al., 2006); the removal of the C-terminal valine in peptain-1 does not affect its functions. In peptain-1 with a cysteine residue at the C-terminus, there are 21 amino acids with an additional cysteine residue at the C-terminus for conjugating Cy5; the addition of a cysteine residue reduces the chaperone activity, but this peptide was used only to determine distribution in retina upon intravitreal injection.

### Animals

All animal procedures complied with the ARVO Statement for the Use of Animals in Ophthalmic and Vision Research and approved by the Institutional Animal Care and Use Committee (Protocol #00239). Both male and female C57BL/6J mice were obtained from Jackson Laboratories (Stock No: 000664, Bar Harbor, ME, USA) or bred in-house with a 12:12-h light/dark cycle with ad libitum food and water.

### Entry of peptain-1 into the retina

Conjugation of peptain-1 containing a cysteine residue at the C-terminus with sulfo-cyanine5 maleimide (Cy5) dye was carried out as previously described (Stankowska et al., 2019). HRECs were treated with 10 μg/mL of peptain-1-Cy5. After 24 h, the cells were fixed with 4% paraformaldehyde for 10 min, and confocal microscopy images were taken (Nikon Eclipse Ti, Nikon instruments Inc., Tokyo, Japan). Mice (12 weeks old) were intravitreally injected with 1 μg of peptain-1-Cy5 in 2 μL of PBS. The contralateral eyes were used as controls. After 4 h, retinas were dissected out and fixed in 4% paraformaldehyde for 1 h. Retinal flat mounts were prepared and transferred to microscope slides, and Cy5 fluorescence intensity was collected using an Odyssey^®^ CLx Imager (LI-COR Biotechnology, Lincoln, NE). After that, the retinas were homogenized, and the fluorescence intensity of the retinal tissue extracts was measured at excitation 647 nm and emission 665 nm with a FluoroMax-4 spectrofluorometer (HORIBA Scientific, Edison, NJ) as previously described (Stankowska et al., 2019). After 24 h of peptain-1-Cy5 injection, the Cy5 fluorescence intensity of blood vessels in the retina was visualized using confocal laser scanning microscopy.

### Proinflammatory cytokine-mediated apoptosis in HRECs

HRECs were isolated, characterized and cultured as previously described (Nahomi et al., 2018) and treated with peptain-1 or scrambled peptides (200 μg/mL) for 3 h in serum-free MEM and then a combination of proinflammatory cytokines [20 ng/mL of IFN-γ (BD Biosciences, San Jose, CA, Cat #285-IF) +TNF-α (Invitrogen, Carlsbad, CA, Cat# PHC3015L) + IL-1β (Invitrogen, Cat #PHC0815)] for 48 h. Cell lysates were prepared with 1X RIPA buffer (Thermo Fisher Scientific, Waltham, MA, Cat# 89900) containing a protease inhibitor cocktail (1:100, Millipore Sigma, Cat# P8340). The protein concentration of the cell lysate was measured by the BCA method, and 25 μg protein was used for immunoblotting, as previously described (Nahomi et al., 2019). The membrane was cut into two halves after electrophoretic transfer at ∼40 kDa, and the half of the membrane below ∼40 kDa was incubated overnight at 4°C with cleaved caspase-3 antibody (Cell Signaling, MA, Cat# 9661, 1:1,000 dilution). The other half of the membrane was treated with β-actin antibody (Cell Signaling, MA, Cat# 4970, 1:10,000 dilution). Membranes were treated with HRP-conjugated goat anti-rabbit IgG (Cell Signaling, MA, Cat# 7074S, 1:1,000 dilution for cleaved caspase-3 and 1:5,000 for β-actin antibody) for 1 h at room temperature. After developing with a SuperSignal™ West Femto Maximum Sensitivity Substrate (Thermo Fisher Scientific, Cat# 34096) or SuperSignal™ West Pico PLUS Chemiluminescent Substrate (Thermo Fisher Scientific, Cat# 34580), membrane that was treated with cleaved-caspase-3 antibody was stripped using Restore Western Blot Stripping Buffer (Thermo Fisher Scientific, Cat# 21059) and probed for peptain-1 antibody (developed in our laboratory, 1:500 dilution) (Nahomi et al., 2019). Densitometric analysis was performed using ImageJ software (NIH). Cell apoptosis was also evaluated via the TUNEL assay. Cells were fixed utilizing 4% paraformaldehyde and permeabilized with 0.25% Triton X-100. Cells were then incubated with biotinylated-dUTP and terminal Transferase. Cells were then incubated with rhodamine Avidin-D and visualized using confocal microscopy. Total cell counts and TUNEL-positive cells were counted manually using ImageJ. Three images were taken from each well and the percentage of TUNEL-positive cells in each well was calculated as the weighted average of these three values.

### Retinal ischemia-reperfusion (I/R) injury in mice

To evaluate the effect of peptain-1 on retinal capillary degeneration, mice were subjected to retinal I/R injury as previously described (Stankowska et al., 2019). Mice were randomly assigned to the peptain-1 treatment group or scrambled peptide group. Mice were intravitreally injected with 0.5 μg of peptain-1 or Scrb-2 peptide in 1 μL of PBS immediately after I/R injury and 7 days later. One microliter of 0.1% DMSO in PBS was injected as vehicle control, and animals were euthanized on day 1, 2 or day 14 post-I/R injury.

### Acellular capillaries in retina

Fourteen days after the initial injection of peptide, mice were sacrificed, and their eyes were enucleated. Retinas were isolated by dissection under light microscopy and treated with elastase (40 U/ml) for 35 min at 37°C with gentle agitation. After careful removal of the internal limiting membrane, retinas were placed in 12-well plates with Tris-HCl buffer, pH 7.8, and shaken overnight to loosen the remaining cells. The retinas were then transferred to glass microscopy slides, and the remaining neuronal tissues were carefully dislodged through gentle agitation produced by a 20 μL pipette in Tris-HCl. Periodic acid–Schiff staining was used to visualize the isolated retinal capillary layer. An inverted fluorescence microscope was used to image the mounted capillaries, and the acellular capillaries from each treatment group were counted and analyzed.

### Whole mount immunostaining of mouse retina

The animals were euthanized one day after I/R injury, and the eyes were enucleated and fixed with 4% paraformaldehyde overnight at 4°C. The following day, the retinas were dissected and washed thrice in PBS. Retinas were then blocked in a solution of 5% normal donkey serum and 1% Triton X-100 in PBS for 1 h. Whole-mounted retinas were immunostained overnight at 4°C with an Iba1 antibody (1:500 dilution, Cat# ab178846, Abcam, Cambridge, MA) to detect activated microglia, followed by a 2-h incubation at room temperature with Texas red-conjugated goat anti-rabbit IgG antibody (1:1000 dilution, Cat# T-2767, Invitrogen). Four fields from mid-peripheral regions of the ganglion cell layers were imaged using a confocal microscope (Nikon ECLIPSE Ti, Japan).

### Quantitative real-time PCR

To measure proinflammatory cytokine levels, mice were sacrificed, and retinas were dissected 2 days after I/R injury. Quantitative real-time PCR was performed as previously described (Nam et al., 2020). Retinal RNA was extracted using QIAzol reagent (Qiagen, Cat# 79306, Valencia, CA, USA) and RNA was purified using the RNeasy Plus Micro Kit (Qiagen, Cat# 74034). Two micrograms of RNA were reverse transcribed to synthesize cDNA using the QuantiTect Reverse Transcription Kit (Qiagen, Cat# 205311). Quantitative real-time PCR was performed with SsoAdvanced™ Universal SYBR^®^ Green Supermix (Cat# 1725271, Bio-Rad, Richmond, CA, USA) using an iCycler iQ5 Real-Time PCR Detection System (Bio-Rad). The sequences of PCR primers were as follows: TNF-α forward primer; 5′- GACAAGGCTGCCCCGACTA-3′, reverse primer; 5′- AGGGCTCTTGATGGCAGAGA –3′, IL-1β forward primer; 5′- GAAATGCCACCTTTTGACAGTG-3′, reverse primer; 5′- TGGATGCTCTCATCAGGACAG-3′, IFN-γ forward primer; 5′- CAGGCCAGACAGCACTCGAATG-3′, reverse primer; 5′- AGGGAAGCACCAGGTGTCAAGT-3′. The mRNA levels were analyzed using the comparative Ct method (2-ΔΔCT) and were normalized to β-actin (forward primer; 5′-AGAAAATCTGGCACCACACC-3′, reverse primer; 5′-GGGGTGTTGAAGGTCTCAAA-3′).

### Statistical analysis

GraphPad Prism software version 10 (GraphPad Prism Software, Inc., San Diego, CA, USA) was used for all statistical analyses. Data are expressed as the means ± standard deviation (SD) of the indicated independent experiments. Tukey’s multiple comparison test was used to determine significant differences among treatment groups. A *p*-value of < 0.05 was considered statistically significant.

## Results

### Peptain-1 protects HRECs against proinflammatory cytokine-mediated apoptosis

**FIGURE 1:**
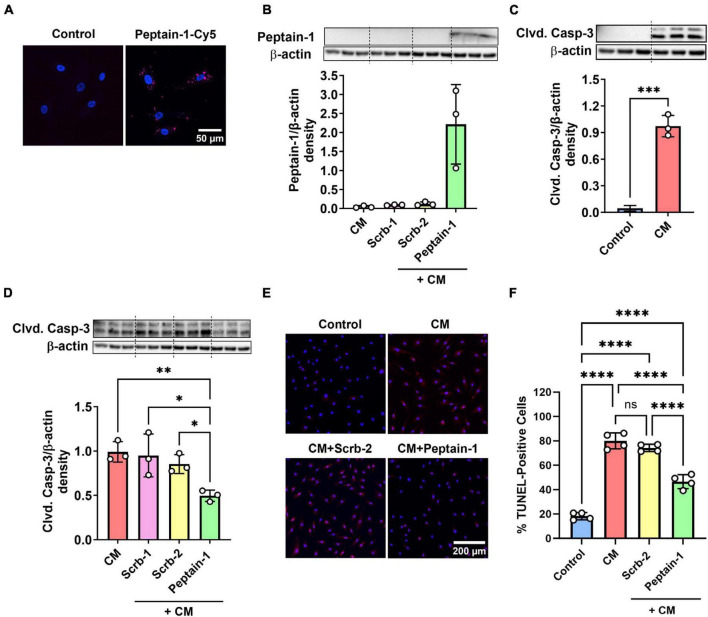
Peptain-1 inhibits proinflammatory cytokine-induced apoptosis in primary human retinal endothelial cells (HRECs). Cy5-conjugated peptain-1 (peptain-1-Cy5) was treated (10 μg/mL) for 24 h and visualized by confocal microscopy, demonstrating that peptain-1 is cell permeable. **(A)** Cells were treated with or without peptide (200 μg/mL) for 3 h and then with a mixture of IFN-γ, TNF-α and IL-1β (20 ng/mL) to induce apoptosis for an additional 48 h. Western blotting demonstrated the presence of peptain-1 in the cell lysates. **(B)** Western blotting showed that cleaved caspase-3 (Clvd. Casp-3) was increased after 48 h of cytokine stimulation, while peptain-1 effectively inhibited this increase. **(C,D)** Beta-actin was used as the loading control in all panels. The graph (densitometry plot) represents the mean ± SD of triplicate measurements. The TUNEL assay was used to assess apoptosis, and representative confocal microscopy images are shown in **(E)**. TUNEL-positive cells were labeled in red, and cell nuclei were labeled with DAPI (blue). The percentage of TUNEL-positive cells in each treatment group is presented in **(F)**. The graph represents the average of TUNEL-positive cells from each well in each treatment group ± SD. Peptain-1 significantly reduced the number of apoptotic cells under inflammatory stress. CM, cytokine mixture and Scrb-1; Scrb-2, scrambled peptide 1 and 2. ns, not significant, *p < 0.05, **p < 0.01, ***p < 0.001, and ****p < 0.0001.

The effect of peptain-1 on the apoptosis of HRECs was evaluated. Our data showed that peptain-1-Cy5 was permeable to HRECs (Figure 1A). Peptain-1 was pre-treated for 3 h, and then a mixture of IFN-γ, TNF-α, and IL-1β (20 ng/mL) was incubated to induce apoptosis (Nahomi et al., 2014). After 48 h, peptain-1 was detected in the cell lysate using Western blotting with a peptain-1 specific antibody (Figure 1B). Cleaved caspase-3 activation in HRECs was measured by western blotting and a significant increase in cleaved caspase-3 levels (21.4-fold, *p* < 0.001) in cells treated with proinflammatory cytokines versus untreated cells was demonstrated (Figure 1C). Similar levels of cleaved caspase-3 in cells treated with either of the scrambled peptides were also observed (Figure 1D). In contrast, cleaved caspase-3 levels were significantly reduced in cytokine mixture (CM) + peptain-1 treated cells when compared to those cells treated with the CM alone (0.5-fold vs. CM, *p* < 0.01) and CM + scrambled peptide treated cells (*p* < 0.05) (Figure 1D). Apoptosis was also assessed using TUNEL staining, and representative images from confocal microscopy are shown in Figure 1E. Treatment with the CM significantly increased the percentage of TUNEL-positive cells compared to control group (4.4-fold, *p* < 0.0001) (Figure 1F). Additionally, the scrambled peptide-treated group did not show a reduction in the percentage of apoptotic cells. However, cells treated with peptain-1 showed a significantly reduced percentage of apoptotic cells in response to the proinflammatory conditions (*p* < 0.0001).

### Intravitreally injected peptain-1 reaches the retina

**FIGURE 2:**
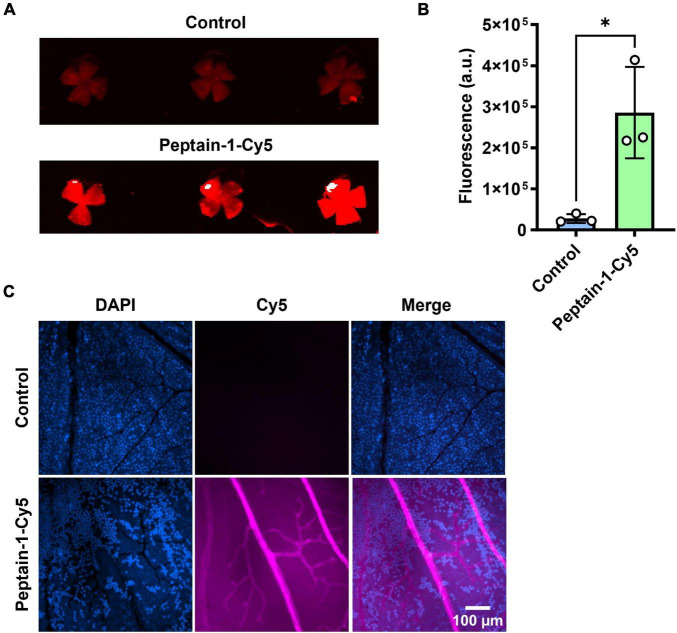
Peptain-1 is permeable to the retinal capillaries. Peptain-1-Cy5 was intravitreally injected (1 μg). Mice were sacrificed four h **(A,B)** and 24 h **(C)** after injection. Fluorescence intensity was detected in the retinal flat mount panel **(A)**, homogenate panel **(B)** and blood vessels panel **(C)** of the injected eye. Uninjected contralateral eyes were used as a control. The nuclei were stained with DAPI (blue). *p < 0.05. a.u, arbitrary units.

The ability of intravitreally injected peptain-1 to enter the retina and retinal blood vessels was then assessed. For this, peptain-1 labeled with Cy5 was intravitreally injected. The accumulation of peptain-1-Cy5 in the retina was seen 4 h after injection (Figure 2A). Similarly, an increase in Cy5 fluorescence in the retinal homogenate of the injected group as compared to the control group was observed (Figure 2B). To assess this in retinal blood vessels, Cy5 fluorescence in retinal flat mounts was imaged using a confocal microscope 24 h after injection. As shown in Figure 2C, fluorescence of peptain-1-Cy5 was visualized throughout the retina and in retinal blood vessels.

### Peptain-1 inhibits retinal capillary degeneration caused by I/R injury

**FIGURE 3:**
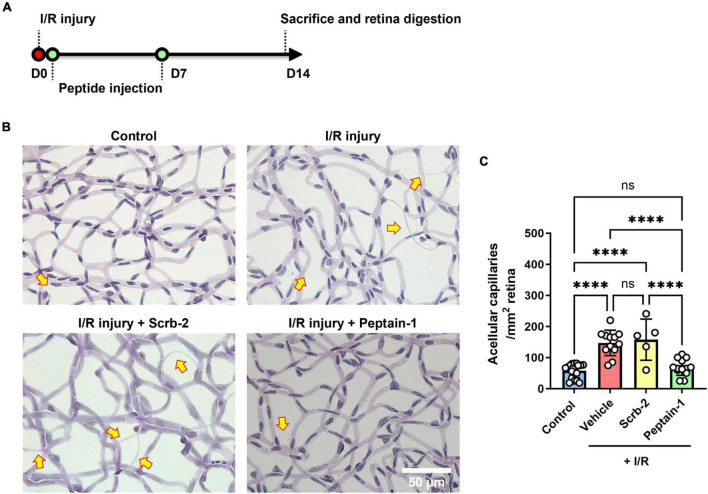
Intravitreal injection of peptain-1 inhibits retinal capillary degeneration following ischemia/reperfusion (I/R) injury. The timeline for peptide injections and I/R injury is shown in **(A)**. Mice were subjected to I/R injury and after 2 weeks, mice were euthanized, and retinas were digested with elastase, and retinal capillaries were stained with periodic acid and Schiff solution. Representative periodic acid–Schiff-stained images were shown in **(B)**, and acellular capillaries were counted **(C)**. Arrows indicate acellular capillaries. The bar graph shows the number of acellular capillaries, which were significantly increased in the retinas of mice subjected to I/R injury followed by injection of either PBS (vehicle) or scrambled peptide (Scrb-2, 0.5 μg) compared to the uninjured contralateral retinas, and was significantly decreased in the retinas of mice treated with peptain-1 (0.5 μg). Data are expressed as the mean ± SD. ns, not significant, ****p < 0.0001, *n* = 5–19. Scale bar = 50 μm.

The protective effects of peptain-1 against retinal I/R injury were then assessed. Scrambled peptide (Scrb-2) or peptain-1 was intravitreally injected immediately after and 7 days after I/R injury (Figure 3A). After 14 days, enzyme-digested retinas showed that retinas with I/R injury followed by vehicle injection had a significantly higher number of acellular degenerated capillaries (2.9-fold, *p* < 0.0001) than contralateral retinas (Figures 3B, C). The scrambled peptide (Scrb-2) treatment showed a similar pattern to the vehicle treatment (2.7-fold, not significant between the vehicle- and scrambled peptide-treated groups). However, the peptain-1-treated retinas showed significantly reduced formation of acellular capillaries (1.2-fold decrease, *p* < 0.0001 vs. vehicle, and *p* < 0.01 vs. Scrb-2 peptide-treated group).

### Intravitreally injected peptain-1 inhibits neuroinflammation in the retina

**FIGURE 4:**
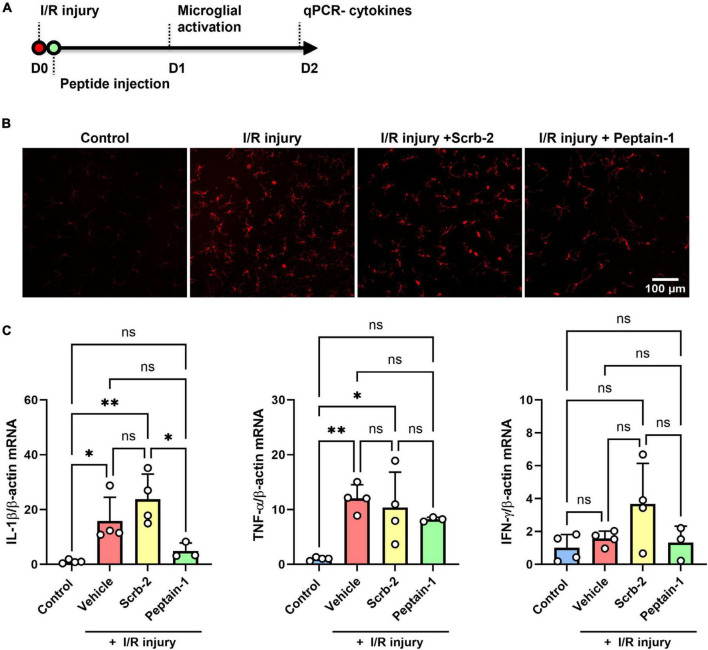
Intravitreal injection of peptain-1 inhibits inflammatory cytokine upregulation and microglial activation in I/R-injured retinas. The timeline for peptide injections and I/R injury is shown in **(A)**. Mice were subjected to I/R injury and injected with 1 μl of PBS alone (vehicle), 0.5 μg peptain-1, or scrambled peptide was intravitreally injected immediately after I/R injury. **(B)** One day after retinal I/R injury, retinas were isolated, whole retinal flatmounts were immunostained for Iba1 (red) to identify activated microglia. The results showed that I/R injury induces microglial activation, but treatment with peptain-1 reduced this activation. **(C)** Two days after I/R injury, mice were euthanized, retinas were dissected out, and total RNA was lysed from the retinas. The mRNA levels of proinflammatory cytokines, IL-1β, TNF-α, and IFN-γ were measured by qPCR. The injection of peptain-1 significantly reduced IL-1β mRNA levels that were induced by I/R injury. Data are expressed as the mean ± SD. ns = not significant, *p < 0.05, **p < 0.01, *n* = 3–4.

Scrambled peptide (Scrb-2) or peptain-1 was intravitreally injected immediately after I/R injury, and the effect of peptain-1 on microglial activation and its inhibitory effect of neuroinflammation were evaluated (Figure 4A). One day after I/R injury, eyes treated with the vehicle or Scrb-2 exhibited microglial activation, as evidenced by hypertrophied and amoeboid morphologies. In contrast, the peptain-1-treated group showed reduced numbers of activated microglia with a more ramified appearance. Interestingly, I/R injury did not significantly change the total number of Iba1-positive cells compared to the control, suggesting the presence of early inflammatory responses. These findings indicate that peptain-1 effectively attenuates acute neuroinflammation following I/R injury (Figure 4B). Subsequently, the effect of peptain-1 on the expression of proinflammatory cytokines *in vivo* was then evaluated. At 2 days after I/R injury, the mRNA levels of IL-1β and TNF-α were significantly increased in the vehicle alone treated retinas (15.8-fold, *p* < 0.05, and 9.0-fold, *p* < 0.01, respectively) compared to control retinas (Figure 4C). Peptain-1 treatment reduced the mRNA levels of IL-1β (4.9-fold vs. 15.8-fold in vehicle group) and TNF-α (7.1-fold vs. 9.0-fold in vehicle group), but the decreases were statistically insignificant. The Scrb-2 peptide-treated retinas unexpectedly showed even higher mRNA levels for IL-1β (23.7-fold, *p* < 0.01) when compared control retinas, and 1.5-fold higher (not significant) when compared to vehicle alone treated retinas after I/R injury. The mRNA levels for TNF-α in the Scrb-2 peptide-treated group were 11.3-fold higher (*p* < 0.05) when compared control retinas, but similar to vehicle alone treated retinas after I/R injury. The mRNA levels of IFN-γ were unchanged in all groups.

## Discussion

The objective of this study was to determine whether peptain-1 can inhibit proinflammatory cytokine-mediated apoptosis of HRECs and retinal capillary degeneration in mice subjected to I/R injury. The impetus for this study was our previous observations that intraperitoneal injection of peptain-1 blocked lens epithelial cell apoptosis and cataract formation in rats and retinal ganglion cell death in rodent models of glaucoma (Nahomi et al., 2013b; Stankowska et al., 2019) and that intravitreally injected peptain-1 blocked retinal ganglion cell death in mouse models of glaucoma (Nam et al., 2022). The ability of peptain-1 to block retinal capillary cells was first verified in cultured HRECs. The results of our study clearly showed that peptain-1 enters HRECs and blocks proinflammatory cytokine-mediated apoptosis. Analysis with the TUNEL assay also supports an overall statistically significant reduction in apoptotic cells in culture. These findings are analogous to our previous findings on cultured RGCs and rat retinal explants, which showed that peptain-1 blocked hypoxia-mediated apoptosis (Stankowska et al., 2019). At this point, it is unclear whether the entry of peptain-1 into HRECs was active or facilitated. One previous study showed that peptain-1 is transported into human RPE cells through a sodium-coupled oligopeptide transporter (Sreekumar et al., 2013). It is possible that a similar transporter in HRECs transported peptain-1 into cells, but that possibility has to be investigated in a future study.

Diabetes induces apoptosis of different cell types in the retina, including capillary pericytes and endothelial cells (Behl et al., 2008; Barber et al., 2011). Pericyte apoptosis is an early event in DR and is followed by apoptosis of endothelial cells, which can lead to increased vascular permeability, macular edema, and retinal angiogenesis (Cunha-Vaz et al., 1975; Hammes et al., 2002; Watson et al., 2017). Various mechanisms, including those mediated by oxidative stress and inflammation, have been proposed as initiators of such changes. In fact, several antioxidants and anti-inflammatory agents, when administered intravitreally or intraperitoneally, have been shown to inhibit retinal changes in diabetes (Cecilia et al., 2019; Rossino and Casini, 2019). However, the precise mechanism by which peptain-1 inhibits retinal endothelial cell death and capillary degeneration has to be investigated in future studies.

AlphaB-crystallin is a robust anti-apoptotic protein; in addition to blocking intracellular apoptotic pathways, such as activation of procaspase-3 and binding to Bax, (Lanneau et al., 2008; Nahomi et al., 2013a; Bakthisaran et al., 2015; Nagaraj et al., 2016) it has been shown to reduce oxidative stress and inflammatory stress (through binding to inflammatory cytokines or suppressing their expression) (Rothbard et al., 2012; Erni et al., 2019; Xu et al., 2019). Many cell types in the retina, including capillary cells, express αB-crystallin (Heise and Fort, 2011). It is noteworthy that the levels of αB-crystallin are upregulated both in experimental diabetes in rodents (Kumar et al., 2005; Fort et al., 2009) and in human diabetic retinas (Rübsam et al., 2018), but one study demonstrated that the chaperone activity of αB-crystallin and its binding ability to proapoptotic proteins are reduced in diabetic retinas (Losiewicz and Fort, 2011). Since the chaperone activity is likely directly related to anti-apoptotic activity [drawing a parallel from the findings on αA-crystallin (Pasupuleti et al., 2010)], the loss of chaperone activity of αB-crystallin could lead to its diminished antiapoptotic activity and contribute to capillary cell death in diabetic retinas. In support of this possibility is our previous finding that overexpression of αB-crystallin inhibits high glucose-induced apoptosis of retinal endothelial cells (Liu et al., 2004). Peptain-1 exhibits chaperone activity (binding to partially denatured proteins and preventing their further denaturation and aggregation) as well as anti-apoptotic activity (Nahomi et al., 2013b). Its anti-apoptotic function is similar to that of αB-crystallin, from which it is derived, as previously reported (Zhou et al., 2014; Sreekumar et al., 2018). Since the loss of αB-crystallin activity is a probable cause for capillary degeneration, it is reasonable to assume that supplementation with peptain-1 could mitigate that deficiency in diabetic retina. It is also possible that peptain-1 is able to offset conditions that favor apoptosis of capillary cells due to the loss of other heat shock proteins (Nahomi et al., 2014). In addition, similar to the parent αB-crystallin, peptain-1 might intercept inflammatory pathways and prevent capillary degeneration.

Several studies showed that the expression of proinflammatory cytokines is upregulated in the diabetic retina (Brucklacher et al., 2008; Joussen et al., 2009; Ucgun et al., 2020). Previous studies demonstrated that I/R injury also induces the upregulation of proinflammatory cytokines such as IL-1β, IL-6, and TNF-α (Wei et al., 2011; Chen et al., 2022). Thus, I/R injury replicates the changes in proinflammatory cytokine levels observed in diabetic retinas. We found that the IL-1β mRNA levels were lower in peptain-1-administered retinas compared to the scramble peptide-administered retinas after I/R injury, supporting a role for peptain-1 in reducing inflammation. However, peptain-1 treatment did not affect the mRNA levels of TNF-α, and neither I/R injury nor peptain-1 treatment affected the mRNA levels of IFN-γ. We measured the mRNA levels 2 days after I/R injury, which reflects the phase after microglial activation and the upregulation of cytokines during gliosis. However, this timing might have led us to miss the peak cytokine levels, which are likely to occur within the first day based on previous reports (Wei et al., 2011; Chen et al., 2022). A time course study will be required to further investigate the effects of peptain-1 on the mRNA and protein levels of proinflammatory cytokines after I/R injury.

There are a number of limitations in the current study, chiefly the use of the I/R model. While retinal ischemia is a key factor in the pathogenesis of DR, blindness resulting from DR arises due to a complex interplay of different factors (Frank, 2004). As such, while our use of the I/R model is useful in determining the effects of peptain-1 against retinal capillary degeneration and could provide key insights into peptain-1’s usefulness in the early stages of DR, its utility in determining the efficacy against the conditions in true DR is limited. Thus, this study should be viewed as proof of concept. In the future, experiments using a mouse model of diabetes will be required to better appreciate the protective role of peptain-1 in DR conditions. The use of an inflammatory cytokine mixture to induce apoptosis in HRECs in the absence of high glucose (Xie et al., 2008) suffers from similar drawbacks and should be viewed as preliminary work. Furthermore, the levels of cytokines we used were higher than those typically found in the eyes of individuals with diabetes and the serum of diabetic mice (Quevedo-Martínez et al., 2021; Farooq et al., 2022; Wang et al., 2024). It is known that retinal capillary pericytes and endothelial cells rely on each other (Chatterjee and Naik, 2012). While we studied the impact of peptain-1 on endothelial cells, we did not assess its effects on pericytes.

Albeit these limitations, our data suggest that peptain-1 prevents capillary degeneration and inflammation in I/R-injured retinas. Whether a similar protective ability extends to diabetic retinas will need to be investigated. If so, peptain-1 could be developed as a therapeutic agent to treat early lesions in diabetic retinopathy, aiming to prevent inflammation and capillary degeneration. Future efforts could also be directed toward developing methods for the sustained release of peptain-1 in the retina for prolonged protection in the diabetic retina.

## Data availability statement

The original contributions presented in this study are included in the article/supplementary material, further inquiries can be directed to the corresponding author.

## Ethics statement

All animal experiments complied with ARVO guidelines for ophthalmic and vision research, which were approved by the Institutional Animal Care and Use Committee (IACUC), University of Colorado, Aurora. The study was conducted in accordance with the local legislation and institutional requirements.

## Author contributions

M-HN: Conceptualization, Investigation, Methodology, Supervision, Writing–original draft, Writing–review and editing. AD: Formal analysis, Investigation, Methodology, Validation, Writing–original draft. RBN: Investigation, Methodology, Writing–original draft. NC: Investigation, Methodology, Writing–original draft. CH: Investigation, Methodology, Writing–original draft. RHN: Conceptualization, Funding acquisition, Project administration, Resources, Validation, Writing–original draft, Writing–review and editing.
